# Knockdown of the MAPK p38 pathway increases the susceptibility of *Chilo suppressalis* larvae to *Bacillus thuringiensis* Cry1Ca toxin

**DOI:** 10.1038/srep43964

**Published:** 2017-03-06

**Authors:** Lin Qiu, Jinxing Fan, Lang Liu, Boyao Zhang, Xiaoping Wang, Chaoliang Lei, Yongjun Lin, Weihua Ma

**Affiliations:** 1 National Key Laboratory of Crop Genetic Improvement and National Centre of Plant Gene Research, Wuhan, China; 2 Hubei Insect Resources Utilization and Sustainable Pest Management Key Laboratory, College of Plant Science and Technology, Huazhong Agricultural University, Wuhan, China

## Abstract

The bacterium *Bacillus thuringiensis* (Bt) produces a wide range of toxins that are effective against a number of insect pests. Identifying the mechanisms responsible for resistance to Bt toxin will improve both our ability to control important insect pests and our understanding of bacterial toxicology. In this study, we investigated the role of MAPK pathways in resistance against Cry1Ca toxin in *Chilo suppressalis*, an important lepidopteran pest of rice crops. We first cloned the full-length of *C. suppressalis* mitogen-activated protein kinase (MAPK) *p38, ERK1*, and *ERK2*, and a partial sequence of *JNK* (hereafter *Csp38, CsERK1, CsERK2* and *CsJNK*). We could then measure the up-regulation of these MAPK genes in larvae at different times after ingestion of Cry1Ca toxin. Using RNA interference to knockdown *Csp38, CsJNK, CsERK1* and *CsERK2* showed that only knockdown of *Csp38* significantly increased the mortality of larvae to Cry1Ca toxin ingested in either an artificial diet, or after feeding on transgenic rice expressed Cry1Ca. These results suggest that MAPK p38 is responsible for the resistance of *C. suppressalis* larvae to Bt Cry1Ca toxin.

Pore-forming toxins (PFT) play an important role in bacterial pathogenesis and the development of pest resistant strains of crops^1^,^2^,^3^. Several previous studies have shown that PFTs such as streptolysin O (*Streptococcus pyogenes*), α-hemolysin (*Escherichia coli*), α-toxin (*Staphylococcus aureus*) and Crystal (Cry) toxin (*Bacillus thuringiensis*) (Bt) have high toxicity to insect pests^4^,^5^,^6^,^7^. Among these, Bt Cry toxins are the most widely used bacterial pesticides^8^.

The use of transcriptomic and proteomic approaches has been a recent advance in investigating the mechanisms underlying host responses to Bt toxins. For example, the non-olfactory, odorant binding protein C12 has been reported to play a role in the resistance of *Tribolium castaneum* larvae to Cry3Ba and Cry23Aa/Cry37Aa toxins^9^. Knockdown of the ATP synthase subunits beta and actin in *Aedes aegypti* larvae increased their susceptibility to Cry11Aa but silencing the heat shock protein caused larvae to become resistant to this toxin^10^. Moreover, transcriptional profiling has demonstrated that cells trigger different survival mechanisms to counteract the effects of non-lethal doses of Bt toxins^11^,^12^,^13^,^14^,^15^.

Besides high throughput approaches, a few studies have focused on analyzing the roles of specific pathways and genes in resistance to Bt toxins. For example, exposure to Cry1Ab and Cry11Aa activated p38 phosphorylation in both *Manduca sexta* and *A. aegypti*, thereby increasing resistance to these toxins^16^. The unfolded protein (UPR) pathway, induced after activation of Mitogen-activated protein kinase (MAPK) p38, has since been implicated in the resistance of *A. aegypti* to Cry11Aa^17^. In *T. castaneum*, the hemolymph protein Apolipophorin-III acts as an immune response protein following challenge by Cry3Ba^18^,^19^. Insect midgut alkaline phosphatase and cadherin, which are generally thought to be Cry toxin receptors, have also been found to be involved in the immune response to Cry toxins^20^,^21^.

The striped rice borer, *Chilo suppressalis*, is a lepidopteran pest that causes major damage to rice crops in Asia^22^,^23^,^24^,^25^. Previous studies have demonstrated that the Cry1Ca toxin is effective against this pest^26^, and that transgenic Bt crops producing Cry1Ca (T1C-19) are resistant to *C. suppressalis*^27^. In this paper we present the results of experiments designed to determine the function of specific *C. suppressalis* MAPK pathway genes in resistance to Cry1Ca. The results suggest that the p38 pathway plays a major role in resistance to Cry1Ca toxin in *C. suppressalis*.

## Results

### Cloning of *Cs*-*p38*, -*JNK*, -*ERK1/2* cDNA sequences and sequence analysis

**Figure 1:**
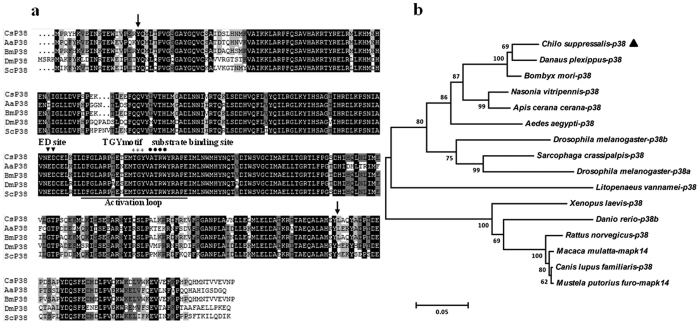
Comparison of *C. suppressalis* p38 (*Cs*p38) with that of other species. (**a**) Multiple-sequence alignment of the deduced amino acid sequence of *Cs*p38 MAPK with other known p38 MAPKs. Amino acids with 100%, 75%, and 50% conservation are shaded in black, dark grey and light grey. The predicted serine/threonine protein kinase (S_TKc) domain was found at position 20–304 (arrow). The characteristic p38 structure, activation loop (underline), substrate binding site (dark spot), ED site (inverted triangle), and TGY motif, are indicated by asterisks. (**b**) Phylogenetic tree of relationships between *Cs*p38 and MAPKs from other species (Supplementary Table S2). The bar indicates a phylogenetic distance of 0.05, the position of *Cs*p38 is indicated by a black triangle.

The full-length of *C. suppressalis p38* cDNA (GenBank accession No.: KU358549) consists of an 82 bp 5′ untranslated region (UTR), a 981 bp 3′ UTR, a TGA terminator sequence, and a 1,080 bp open reading frame (ORF) encoding 360 amino acid residues with a molecular mass of 41.58 kDa and a pI value of 5.84. Pair-wise and multiple sequence alignments revealed that *Cs*p38 contains an activation loop structure, a conserved Thr-Gly-Tyr (TGY) phosphorylation motif, the substrate binding site Ala-Thr-Arg-Trp (ATRW), and the ERK docking (ED) site (Fig. 1a). *Cs*p38 protein was most similar to that in *Bombyx mori* (95.8%), followed by *A. aegypti* (83.5%), *Sarcophaga crassipalpis* (79.3%) and *Drosophila melanogaster* (76.5%). The predicted serine/threonine protein kinase (S_TKc) domain was found at position 20–304 (Fig. 1a). To assess the evolutionary relationship between *Cs*p38 and its homologs, a phylogenetic tree was constructed using the neighbor-joining method based on the amino acid sequences of p38 from selected species. This showed that *Cs*p38 was most homologous to that of *Danaus plexippus* and *B. mori,* which collectively comprised a relatively distinct clade (Fig. 1b).

**Figure 2:**
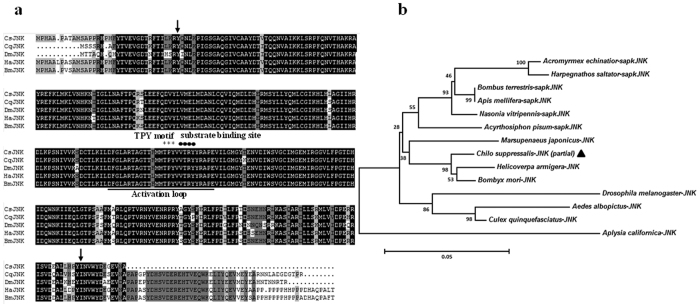
Comparison of *C. suppressalis* JNK (*Cs*JNK) with that of other species. (**a**) Amino acids with 100%, 75%, and 50% conservation are shaded in black, dark grey and light grey. The characteristic JNK structure, activation loop (underline), substrate binding site (dark spot), and TPY motif (asterisks) are found at position 35–331 in the S_TKc domain (arrow). (**b**) Phylogenetic tree of relationships between *Cs*JNK and MAPKs from other species (Supplementary Table S2). The bar indicates a phylogenetic distance of 0.05, the position of *Cs*JNK is indicated by a black triangle.

In this study, we used RT-PCR and rapid amplification of cDNA ends (RACE) technology to clone *C. suppressalis* c-Jun N-terminal kinase *(JNK)* from midgut of 3^rd^ instar larvae. We did not, however, obtain the full-length of this gene. The 1,392 bp partial *CsJNK* cDNA (GenBank accession number: KU358550) we obtained contains a 400 bp 5′ UTR and a partial ORF encoding a predicted protein of 331 amino acids. Multiple sequence alignments showed that *Cs*JNK includs an activation loop structure and a conserved Thr-Pro-Tyr (TPY) phosphorylation motif (Fig. 2a). Its identity with its homologs in *B. mori, Helicoverpa armigera, Culex quinquefasciatus* and *D. melanogaster* is 97.7%, 97.4%, 93.4% and 88.8%, respectively. The predicted S_TKc domain was found at position 20–304 (Fig. 2a). The associated phylogenetic tree shows that *C. suppressalis* JNK clusters with other lepidopteran JNK, and is most closely related to that of *B. mori* and *H. armigera* (Fig. 2b).

**Figure 3:**
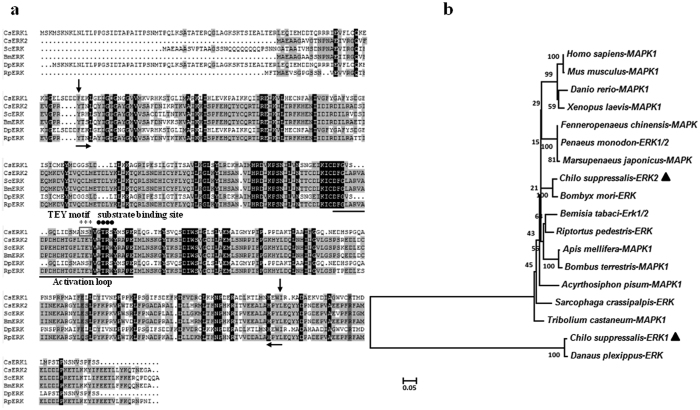
Comparison of *C. suppressalis* ERK1 and ERK2 MAPK genes (*Cs*ERK1 and *Cs*ERK2) with known ERK MAPKs from other species. (**a**) Amino acids with 100%, 75%, and 50% conservation are shaded in black, dark grey and light grey. The predicted S_TKc domain was found at position 90–369 (arrow) of *Cs*ERK1 and 28–316 (arrow) of ERK2. The characteristic ERK structure, activation loop (underline), substrate binding site (dark spot), and TEY motif, are indicated by asterisks. (**b**) Phylogenetic tree of relationships between *Cs*ERKs and MAPKs from other species (Supplementary Table S2). The bar indicates a phylogenetic distance of 0.05, the position of *Cs*ERK is indicated by a black triangle.

The full length of *C. suppressalis* extracellular signal-regulated kinase 1 *(ERK1)* (GenBank accession number: KU358551) cDNA is 1,892 bp and contains a 164 bp 5′ UTR and a 516 bp 3′ UTR. The ORF is 1,209 bp encoding 403 amino acid proteins with a calculated molecular weight of about 44.51 kDa and a PI value of 6.34. The full-length of *CsERK2* (GenBank accession No.: KU358552) is 2,015 bp. The ORF encodes 364 amino acids, the calculated molecular weight is about 41.96 kDa and the estimated PI was 6.09. The predicted S_TKc domain is shown in Fig. 3a. Alignment of *Cs*ERK1 and *Cs*ERK2 with ERK proteins of other species shows that *Cs*ERK2 includes an activation loop structure and a conserved Thr-Glu-Tyr (TEY) phosphorylation motif, and a substrate binding site (Fig. 3a). However, the predicted protein of *Cs*ERK1 has no typical conserved motif. Phylogenetic analysis indicates that *Cs*ERK2 has relatively highest identity with that of *B. mori* (95.1%) and lowest with that of *D. plexippus* (21.8%), whereas *Cs*ERK1 has highest identity with that of *D. plexippus* (98.0%) and lowest with that of *B. mori* (21.2%). Moreover, *Cs*ERK1 and *Cs*ERK2 has very low identity with each other (21.5%) (Fig. 3a). The associated phylogenetic tree places *Cs*ERK1 in a distinct clade with that of *D. plexippus* and that *Cs*ERK2 is most homologous to that of *B. mori* (Fig. 3b).

### Induction of *Csp38, CsJNK* and *CsERK1/2* by Cry1Ca toxin

**Figure 4:**
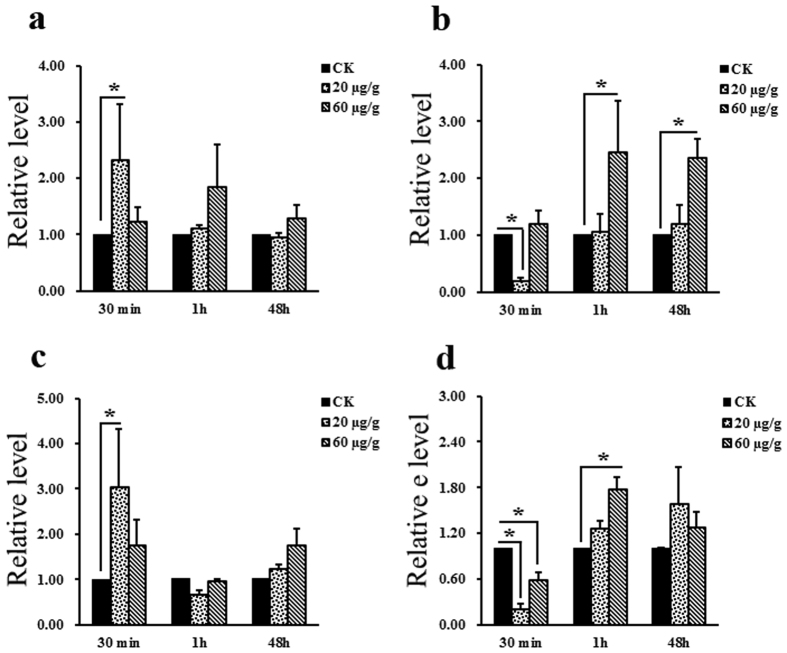
Effects of Cry1Ca toxin on expression of *C. suppressalis* MAPK genes (*Cs*MAPK). (**a**) Estimates of relative *Csp38* transcription levels determined by qRT-PCR of *C. suppressalis* larvae sampled at 30 min, 1 h and 48 h after ingesting Cry1Ca toxin. (**b**) Estimates of relative *CsJNK* transcription levels determined by qRT-PCR of *C. suppressalis* larvae sampled at 30 min, 1 h and 48 h after ingestion of Cry1Ca toxin. (**c**) Estimates of relative *CsERK1* transcription levels determined by qRT-PCR of *C. suppressalis* larvae sampled at 30 min, 1 h and 48 h after ingestion of Cry1Ca toxin. (**d**) Estimates of relative *CsERK2* transcription levels determined by qRT-PCR of *C. suppressalis* larvae sampled at 30 min, 1 h and 48 h after ingestion of Cry1Ca toxin. Relative levels of gene transcription were normalized to that of *EF1* (the internal reference). Asterisks indicate *P* values < 0.05 (ANOVA).

In order to determine whether *Csp38, CsJNK* and *CsERK1/2* were activated by Cry1Ca toxin, we quantified expression of these genes at different periods of time after *C. suppressalis* larvae had ingested this toxin. According to a preliminary dosage screening, we chose the dosages of 20 and 60 μg of final Cry1Ca digested product (FDP) to each gram of artificial food to induce MAPK gene expressions. Ingestion of a diet containing of 20 μg of FDP to each gram of artificial food was followed by a 2-fold increase in *Csp38* expression within 30 min compared to a control group fed on a diet comprised of the usual artificial food plus water (Fig. 4a). A 3-fold Up-regulation of *CsERK1* also occurred within 30 min of ingesting a diet containing 20 μg of FDP to each gram of artificial food (Fig. 4c). *CsJNK* transcription increased after 1 h and 48 h of consuming artificial diet containing 60 μg of FDP to each gram of artificial food (Fig. 4b). Slight alteration of *CsERK2* expression was observed within 1 h of feeding on artificial diet containing 60 μg of FDP to each gram of artificial food (Fig. 4d). In contrast, down-regulation of the *CsJNK* and *CsERK2* transcription were observed within 30 min of feeding on a diet containing 20 μg of FDP to each gram of artificial food (Fig. 4b,d), and down-regulation of the *CsERK2* gene expression was induced within 30 min of feeding on a diet containing 60 μg of FDP to each gram of artificial food (Fig. 4d).

### RNA interference (RNAi) knockdown of *Csp38* caused increased susceptibility to Cry1Ca

**Figure 5:**
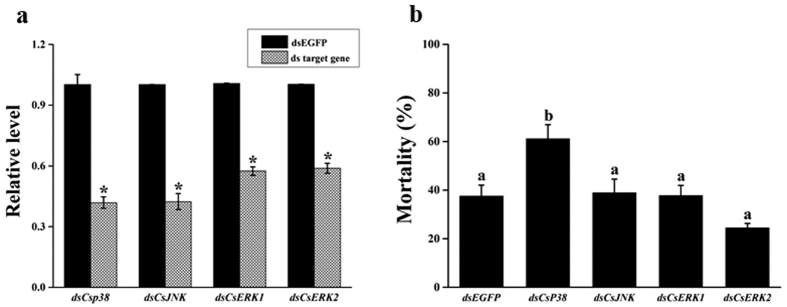
Effect of RNA interference knockdown of *p38, JNK, ERK1* and *ERK2* transcripts on the resistance of *C. suppressalis* larvae to Cry1Ca toxin. (**a**) Estimates of target gene transcription as determined by qRT-PCR and normalized to the expression of a reference gene (*EF1*). qRT-PCR was performed 48 h after *C. suppressalis* neonate larvae had fed on an artificial diet mixed with either *EGFP* dsRNA (the control), or target gene dsRNA. Asterisks indicate *P* values < 0.05 (Student’s t-test). (**b**) Larval mortality. Larvae were first fed their usual artificial food with the addition of either *EGFP* dsRNA (the control), or target gene dsRNA, then their usual artificial food mixed with FDP (30 μg FDP to each gram of food). Corrected mortality was calculated from 5 replicates. Bars with different letters are significantly different (*P* < 0.05; ANOVA).

We used RNAi to test the roles of *Cs*p38, *Cs*JNK, *Cs*ERK1 and *Cs*ERK2 in resistance to Cry1Ca toxicity in *C. suppressalis* larvae. Expression of *Csp38, CsJNK, CsERK1* and *CsERK2* significantly decreased in larvae in which these target genes had been knocked down 48 h compared to a control diet containing *EGFP* dsRNA (Fig. 5a). dsRNA knockdown of *Csp38* led to a significant increase in mortality (61.1%) compared to that in the *EGFP* dsRNA control (37.5%). In contrast, knockdown of *CsJNK, CsERK1* and *CsERK2* did not result in a significant increase in mortality relative to the control (Fig. 5b).

**Figure 6:**
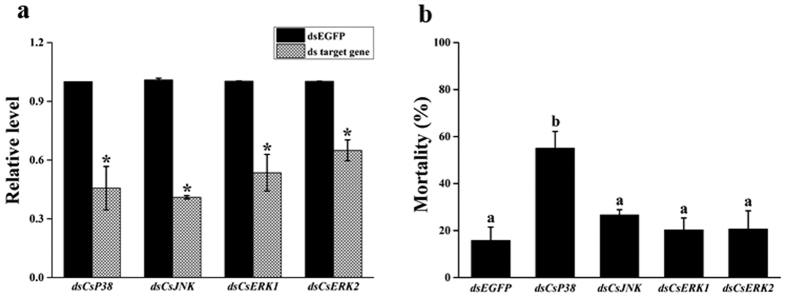
Effect of RNA interference knockdown of *p38, JNK, ERK1* and *ERK2* transcripts on the susceptibility of *C. suppressalis* larvae to Cry1Ca toxin expressed in transgenic rice. (**a**) Estimates of target gene transcription as determined by qRT-PCR and normalized to the expression of a reference gene (*EF1*). qRT-PCR was performed 48 h after *C. suppressalis* neonate larvae had fed on non-transgenic rice (Minghui 63) treated with either *EGFP* dsRNA (the control), or target gene dsRNA. Asterisks indicate *P* values < 0.05 (Student’s t-test). (**b**) Larval mortality. Larvae were first fed on non-transgenic rice (Minghui 63) treated with either *EGFP* dsRNA (the control), or target gene dsRNA, then transgenic rice expressing the Cry1Ca toxin. Corrected mortality was estimated from 5 replicates. Bars with different letters are significantly different (*P* < 0.05; ANOVA).

dsRNA knockdown of *Csp38, CsJNK, CsERK1* and *CsERK2* also caused a significant reduction in the transcription of these genes compared to the *EGFP* dsRNA control (Fig. 6a). Mortalities in the *Csp38, CsJNK, CsERK1* and *CsERK2* knockdown treatment groups after feeding on transgenic rice was 55.1%, 26.7%, 20.3% and 20.6%, respectively. However, only the *Csp38* knockdown treatment group had significantly higher mortality (55.1%) than the control (15.8%) (Fig. 6b).

## Discussion

MAPK signaling pathways are comprised of serine-threonine protein kinases that regulate a variety of cellular processes^28^,^29^,^30^. Multicellular organisms have three subfamilies of MAPKs, including ERK, JNK and p38 MAPKs^31^. Among these, the p38 pathway is the most important in regulating resistance to PFTs.

Since the activation of the p38 pathway by Cry5B toxin in *Caenorhabditis elegans* was first described^2^, several studies have demonstrated that low doses of other PFTs (e.g., aerolysin, pneumolysin, streptolysin and a-hemolysin) can induce the activation of this pathway in cultured-epithelial cell lines^2^,^32^. Similar responses have also been observed in insects, for example, both *M. sexta* and *A. aegypti* were found to activate p38 phosphorylation to protect themselves against Cry toxins^16^. As expected, our results also demonstrate that p38 plays a key role in resistance to Cry1Ca in *C. suppressalis*. This may be related to K^+^ transmission; it has been shown that K^+^ efflux throughout the toxin pore is likely to activate the p38 defensive response to α-toxin, cytolysin or hemolysin^33^. Besides, the different extend of the induced *Csp38* up-regulation between diet mixed with toxin and transgenic rice might be led by different amount of Cry1Ca in artificial diet and transgenic rice.

We also investigated the role of the JNK and ERK pathways in resistance to Cry1Ca toxin in *C. suppressalis*. Although low doses of Cry1Ca induced *csJNK* and *csERK* expression (Fig. 4), no significant difference in mortality were observed between control larvae and those in which *CsJNK* and *CsERK* had been knocked down with RNAi (Figs 5 and 6). We conclude therefore that the JNK and ERK pathways are not involved in resistance to Cry1Ca in *C. suppressalis*. Besides, we did not obtain the full length of *CsJNK*, the sequencing data was always noised in the 3 end part, which may be caused by the secondary structure at the 3′ terminal region of *CsJNK* RNA.

Cellular responses to Cry toxins are complex and require further intensive research. The results of this study show that knockdown of *p38*, but not other MAPK genes, significantly increased the mortality of *C. suppressalis* larvae following ingestion of Cry1Ca. This suggests that p38 is responsible for resistance to Cry1Ca in *C. suppressalis*, and potentially other insect pests. Future work should focus on searching for genes downstream of the p38 pathway that play a role in PFT resistance.

## Materials and Methods

### Insect rearing and Cry1Ca toxin

The founder population of *C. suppressalis* larvae were collected in Dawu County, Hubei Province, China in 2012 and propagated in a laboratory for 4 years. Larvae were kept at 28 ± 1 °C under a 16-h photoperiod, > 80% relative humidity and fed on an artificial diet^34^. The Cry1Ca toxin used in this study is a gift from Dr. Jie Zhang (Institute of Plant Protection, China Academy of Agricultural Science). The toxin is a protein crude extract from a genetic modified *Bacillus thuringiensis* strain, in which Cry1Ca is the only toxin it expressed. The crude extraction product was then digested with trypsin without purification. The FDP was used directly in all bioassays.

### Cloning of three MAPK genes

Total RNA was isolated from actively feeding 4^th^ instar *C. suppressalis* larvae using RNAiso reagent (TaKaRa, Dalian, China). Contaminating genomic DNA was eliminated with RNase-free DNase, and the RNA preparation was then subjected to reverse transcription using a PrimeScript^TM^ RT reagent Kit (TaKaRa, China) according to the manufacturer’s instructions. Partial *p38, JNK, ERK1* and *ERK2* cDNA sequences had been obtained from the *C. Suppressalis* transcriptome determined in previous studies^35^. A 5′ and 3′ RACE kit (TaKaRa, Dalian, China) was used to amplify full-length MAPK genes from *C. Suppressalis* larvae, and pairs of gene-specific and degenerate primers were designed based on the partial sequences using Primer 5.0 software (Supplementary Table S1). PCR products were subcloned into a PMD (18)-T vector (Takara, Dalian, China) and sequenced by the Nanjing Genscript Company, China.

### Sequence analysis

Sequence analysis was conducted on full-length cDNA using an ORF finder tool (http://www.ncbi.nlm.nih.gov/gorf/gorf.html). Sequence alignment was performed with DNAMAN software and phylogenetic analysis conducted with MEGA4.0^36^,^37^. The molecular weight and pI were calculated using the Compute pI/Mw (http://us.expasy.org/tools/pi_tool.html). Phosphorylation sites were determined using the NetPhos 2.0 Server (http://www.cbs.dtu.dk/services/NetPhos/) and protein domains or motifs identified using SMART software (http://smart.embl-heidelberg.de).

### RNAi knockdown of *Csp38, CsJNK*, and *CsERK1/2*

The partial *Csp38, CsJNK*, and *CsERK1/2* sequences were cloned into pET-2P vectors with flanking T7 promoter and T7 terminator sites to produce *Csp38, CsJNK*, and *CsERK1/2* dsRNA^38^. The plasmid pET-2P/*EGFP* was used to produce *EGFP* dsRNA for the control group^38^. Briefly, dsRNA fragments were amplified from the cDNA of *C. suppressalis* larvae with PrimeSTAR HS DNA polymerase (TaKaRa Bio Inc., Dalian, China). PCR products were digested with restriction enzymes (see Supplementary Table S1) and ligated into the previously digested pET-2P. The correct inserts of the recombinant plasmid were confirmed by sequencing by the Genscript Biology Company, Nanjing, China. Plasmids were transformed into *Escherichia coli* HT115 (DE3) competent cells and individual colonies were cultured at 37 °C in 500 ml of LB medium and induced to generate dsRNA with 0.4 mM isopropyl-D-thiogalactoside (IPTG). dsRNA was extracted from aliquots of bacteria with a recently described protocol^39^,^40^.

All subsequent experiments were conducted at 27 °C. To silence RNA, newly hatched larvae were fed a diet comprised of 30 μg of *Cs*-target genes dsRNA, or *EGFP* dsRNA as a control, to each gram of their usual artificial food. Larvae were allowed to feed on this diet for 48 h, then transferred to wells in a 6-well plate where they were fed a diet containing 30 μg FDP toxin to each gram of their usual artificial food (the approximate LC25 determined in a pilot study) for 7 days. The control was the usual artificial food mixed with water. Five replicates were carried out using a total of 120 larvae per treatment. The toxicity to larvae of transgenic rice expressing Cry1Ca toxin was also determined^41^. Newly hatched larvae were fed non-transgenic rice (Minghui 63) smeared with purified dsRNA on the surface of rice plants for 48 h, then transferred to feed on transgenic Cry1Ca rice plants for 7 days. Each treatment was replicated 5 times as described above.

### Real-time quantitative PCR

To examine the effect of Cry toxin on MAPK activation, ten larvae midguts from each treatment were sampled after 30 min, 1 h, and 48 h of FDP (20 μg/g, 60 μg/g) challenge. RNAi knockdown of *Cs-p38*, -*JNK*, -*ERK1* and *Cs-ERK2* was conducted using qRT-PCR with three replicates for each treatment. Total RNA was extracted from larvae and cDNA synthesized using a PrimeScript RT reagent kit with gDNA eraser (perfect real time) (Takara, Dalian, China) according to the manufacturer’s instructions. Gene-specific primers (Supplementary Table S1) were designed by NCBI profile Server (http://www.ncbi.nlm.nih.gov/tools/primer-blast) for qRT-PCR. The *C. suppressalis* elongation factor-1 (*EF-1*) gene was used as the internal reference^42^,^43^. The standard qPCR protocol was as follows: denaturing at 95 °C for 30 sec, followed by 40 cycles of 95 °C for 10 sec and 59 °C for 30 sec. Real-time quantitative PCR was performed in triplicate for each sample using the SYBR^®^ Premix Ex Taq™ (TaKaRa, Dalian, China) and a Bio-rad Detection iQ2 System. Melting curve analysis was performed from 55 °C to 95 °C to determine the specificity of qPCR primers. To determine the efficiency of the qRT-PCR primers, a 5-fold dilution series of 3rd instar larvae cDNA corresponding to one microgram total RNA was used to produce a standard curve (cDNA concentration vs. Ct) with efficiencies calculated from the slope using linear regression. The corresponding qRT-PCR efficiencies were calculated according to the equation: E = (10^[−1/slope]^ − 1)*100^44^,^45^ (Supplementary Table S3).

### Data analysis

Gene expression data were analyzed with the 2^−ΔΔCt^ method^44^. Means and variances of treatments were analyzed with a one-way ANOVA implemented in the SPSS program for windows (SPSS, Chicago, IL, USA).

## Additional Information

**How to cite this article:** Qiu, L. *et al*. Knockdown of the MAPK p38 pathway increases the susceptibility of *Chilo suppressalis* larvae to *Bacillus thuringiensis* Cry1Ca toxin. *Sci. Rep.* **7**, 43964; doi: 10.1038/srep43964 (2017).

**Publisher's note:** Springer Nature remains neutral with regard to jurisdictional claims in published maps and institutional affiliations.

## Supplementary Material

Supplementary Information
