# Supplementary material for: Knockdown of the MAPK p38 pathway increases the susceptibility of Chilo suppressalis larvae to Bacillus thuringiensis Cry1Ca toxin
Source: Sci Rep. 2017 Mar 6;7:43964. doi: 10.1038/srep43964 (PMC5338291; doi:10.1038/srep43964)
Supplement: Supplementary Information [file srep43964-s1.pdf]

SUPPLEMENTARY INFORMATION

Knockdown of the MAPK p38 pathway increases the susceptibility of *Chilo suppressalis* larvae to *Bacillus thuringiensis* Cry1Ca toxin

Lin Qiu[1, 2], Jinxing Fan[2], Lang Liu[2], Boyao Zhang[2], Xiaoping Wang[2], Chaoliang Lei[2], Yongjun Lin[1] & Weihua Ma[1, 2]*

[1]National Key Laboratory of Crop Genetic Improvement and National Centre of Plant Gene Research, Wuhan, China;

[2]Hubei Insect Resources Utilization and Sustainable Pest Management Key Laboratory, College of Plant Science and Technology, Huazhong Agricultural University, Wuhan, China;

* Corresponding author: Weihua Ma

Email: weihuama@mail.hzau.edu.cn

Tel: +86-27-87287207

Fax: +86-27-87287207

Table S1

Specific primers used in the experiments.

Primer Name	Orientation	Primer sequence (5'-3')
		**5'RACE**
P38-GSP1	Antisense	TGTGTTGAGGGTGAGGTTTGAACTCCAC
P38-GSP2	Antisense	GACCGCAAGGGGGTTGGCACCTCGGAAGA
JNK-GSP1	Antisense	CTTCGCCACTATGTTGGACGGCTTCAGA
JNK-GSP2	Antisense	TTCCCCATAAATGCCCAGTGGTTATTGTCAG
ERK1-GSP1	Antisense	GCCAGTGTCCTCACTGATTGTTCCGCCC
ERK1-GSP2	Antisense	CACCTTCTCGGCGTCCGCTTTGCGTATC
ERK2-GSP1	Antisense	AGTCGCAAATCTTCAAATCGCAGGTGGTG
ERK2-GSP2	Antisense	ACAACCATCCCATAGGCTCCTTCCCCAAT
		**3'RACE**
P38-GSP1	Sense	GGTGCCAACCCCCTTGCGGTCAACTTGC
P38-GSP2	Sense	AGAGCTTCGAGGACATGGACCTGCCCGT
JNK-GSP1	Sense	GGAGTTCCAAGATGTGTACCTGGTGATG
JNK-GSP2	Sense	CTGAAGCCGTCCAACATAGTGGCGAAGAGT
ERK1-GSP1	Sense	ATGGATACGCAAAGCGGACGCCGAGAAGG
ERK1-GSP2	Sense	AAAGAGCGGGCGGAACAATCAGTGAGGAC
ERK2-GSP1	Sense	AGTTGGACGACCTGCCGAAGGAGACTCTT
ERK2-GSP2	Sense	GCTGTCTTCGATGACTTCGCCCCACGGC
		**Performing the qPCR**
EF-1-F	Sense	TGAACCCCCATACAGCGAATCC
EF-1-R	Antisense	TCTCCGTGCCAACCAGAAATAGG
P38-F	Sense	TCATTTACGCGGTTTTGAGTGCAA
P38-R	Antisense	TGCAAACTGTCGATCGCTGAA
JNK-F	Sense	GGCAGCTACGATACTGGCAT
JNK-R	Antisense	ACTCCCGATGTTTCTGCGTT
ERK1-F	Sense	TGCCTGCCTATATTTCGGCAACTT
ERK1-R	Antisense	CCGGTGGAAGGGTGAGGTTC
ERK2-F	Sense	CTGCGTCTGTACGGGAGTGTC
ERK2-R	Antisense	CGAACAGTTATTTGCCACCAGAAGT
		**Primers for dsRNA Synthesizing**
ds*Cs*p38-F		atGAATTCCGAACAGGCACATTAACTTCGGAA
ds*Cs*p38-R		atGGTACCTTTCGAAAATTCGTTCGTCCGAA
ds*Cs*JNK-F		atGAATTCACAGTGACGTAGAGTG
ds*Cs*JNK-R		taGAATTCTACGCCGCGCATACGATACCCTGT
ds*Cs*ERK1-F		atGAATTCTGAAGAAGAATCCTGACGAAAGAG
ds*Cs*ERK1-R		atGGTACCTGGCGAGAAATGAAACTAACG
ds*Cs*ERK2-F		atcGAATTCAAGGAGACTCTTAAGAAGTACA
ds*Cs*ERK2-R		tagGAATTCTATGTAATCTCGTTGAGAGAGAACT

Table S2

Protein sequences of MAPKs used to construct phylogenetic tree.

Code name	Organism	Accession number
	**P38**	
Bmp38	*Bombyx mori*	NP_001036996.1
Dpp38	*Danaus plexippus*	EHJ76051.1
Lvp38	*Litopenaeus vannamei*	AGG82488.1
Dmp38b	*Drosophila melanogaster*	NP_477361.1
Nvp38	*Nasonia vitripennis*	NP_001136337.1
Acp38	*Apis cerana* cerana	ADT91683.1
Scp38	*Sarcophaga crassipalpis*	BAF75366.1
Aap38	*Aedes aegypti*	XP_001653240.1
Dmp38a	*Drosophila melanogaster*	AAC39031.1
Xlp38	*Xenopus laevis*	BAI67112.1
Drp38	*Danio rerio*	BAB11808.1
Rnp38	*Rattus norvegicus*	AAB51285.1
Mmmapk14	*Macaca mulatta*	AFI33384.1
Clp38	*Canis lupus* familiaris	AAC36131.1
Mpmapk14	*Mustela putorius* furo	AES01588.1
	**JNK**	
NvsapkJNK	*Nasonia vitripennis*	XP_008212489.1
AaJNK	*Aedes albopictus*	AAO31950.1
CqJNK	*Culex quinquefasciatus*	XP_001842827.1
DmJNK	*Drosophila melanogaster*	AAB97094.1
AcJNK	*Aplysia californica*	NP_001191547.1
MjJNK	*Marsupenaeus japonicus*	BAI87826.1
HaJNK	*Helicoverpa armigera*	AEE81067.1
BmJNK	*Bombyx mori*	NP_001103396.1
ApsapkJNK	*Acyrthosiphon pisum*	XP_001945460.2
AesapkJNK	*Acromyrmex echinatior*	EGI62749.1
HssapkJNK	*Harpegnathos saltator*	EFN90159.1
BtsapkJNK	*Bombus terrestris*	XP_003402842.1
AfsapkJNK	*Apis mellifera*	XP_392806.3
	**ERK**	
BmERK	*Bombyx mori*	NP_001036921.1
BtERK1/2	*Bemisia tabaci*	AEE81046.1
Ammapk1	*Apis mellifera*	XP_393029.2
PmERK1/2	*Penaeus monodon*	ADT80930.1
Mjmapk	*Marsupenaeus japonicus*	BAH86598.1
Tcmapk1	*Tribolium castaneum*	XP_966833.1
Btmapk1	*Bombus terrestris*	XP_003394197.1
Apmapk1	*Acyrthosiphon pisum*	XP_001952106.1

Hsmapk1	*Homo sapiens*	NP_002736.3
Drmapk1	*Danio rerio*	NP_878308.2
Xlmapk1	*Xenopus laevis*	NP_001083548.1
Fcmapk	*Fenneropenaeus chinensis*	AHA83424.1
Mmmapk1	*Mus musculus*	NP_001033752.1
DpERK	*Danaus plexippus*	EHJ76872.1
ScERK	*Sarcophaga crassipalpis*	BAF75365.1
RpERK	*Riptortus pedestris*	BAN20448.1

Table S3

Primer specifications for optimized qRT-PCR amplification of *Chilo suppressalis*.

**Gene name**	**Efficiency**	**R^2**	**Slope**
CsEF1	99.8%	0.992	-3.326
Csp38	100.9%	0.994	-3.300
CsJNK	103.9%	0.995	-3.235
CsERK1	99.9%	0.992	-3.325
CsERK2	103.5%	0.980	-3.242
